# Anti-Microbiota Vaccine Reduces Avian Malaria Infection Within Mosquito Vectors

**DOI:** 10.3389/fimmu.2022.841835

**Published:** 2022-03-03

**Authors:** Justė Aželytė, Alejandra Wu-Chuang, Rita Žiegytė, Elena Platonova, Lourdes Mateos-Hernandez, Jennifer Maye, Dasiel Obregon, Vaidas Palinauskas, Alejandro Cabezas-Cruz

**Affiliations:** ^1^Nature Research Centre, Akademijos 2, Vilnius, Lithuania; ^2^ANSES, INRAE, Ecole Nationale Vétérinaire d’Alfort, UMR BIPAR, Laboratoire de Santé Animale, Maisons-Alfort, France; ^3^SEPPIC Paris La Défense, La Garenne Colombes, France; ^4^School of Environmental Sciences, University of Guelph, Guelph, ON, Canada

**Keywords:** anti-microbiota vaccines, avian malaria, mosquitoes, networks, microbiota

## Abstract

Animal and human pathogens that are transmitted by arthropods are a global concern, particularly those vectored by mosquitoes (e.g., *Plasmodium* spp. and dengue virus). Vector microbiota may hold the key to vector-borne pathogen control, as mounting evidence suggests that the contributions of the vector microbiota to vector physiology and pathogen life cycle are so relevant that vectorial capacity cannot be understood without considering microbial communities within the vectors. Anti-tick microbiota vaccines targeting commensal bacteria of the vector microbiota alter vector feeding and modulate the taxonomic and functional profiles of vector microbiome, but their impact on vector-borne pathogen development within the vector has not been tested. In this study, we tested whether anti-microbiota vaccination in birds targeting Enterobacteriaceae within mosquito midguts modulates the mosquito microbiota and disrupt *Plasmodium relictum* development in its natural vector *Culex quinquefasciatus*. Domestic canaries (*Serinus canaria domestica*) were experimentally infected with *P. relictum* and/or immunized with live vaccines containing different strains of *Escherichia coli*. Immunization of birds induced *E. coli*-specific antibodies. The midgut microbial communities of mosquitoes fed on *Plasmodium*-infected and/or *E. coli*-immunized birds were different from those of mosquitoes fed on control birds. Notably, mosquito midgut microbiota modulation was associated with a significant decrease in the occurrence of *P. relictum* oocysts and sporozoites in the midguts and salivary glands of *C. quinquefasciatus*, respectively. A significant reduction in the number of oocysts was also observed. These findings suggest that anti-microbiota vaccines can be used as a novel tool to control malaria transmission and potentially other vector-borne pathogens.

## Introduction

Mosquitoes are vectors of major human diseases such as dengue (caused by dengue virus), and malaria (caused by *Plasmodium* spp.) (1). According to literature, there are more than 50 avian *Plasmodium* species and new species are discovered every year (2) and infection by these parasites is common in some bird species (3). Among the *Plasmodium* species affecting birds, *P. relictum* is listed among the most invasive organisms in the world, infecting more than 300 bird species and is prevalent all around the world (4). *Plasmodium relictum* is transmitted mostly by *Culex* mosquitoes, including *Culex pipiens* and *Culex quinquefasciatus* (5). The midgut is the first organ in which *P. relictum* ingested with the host blood can survive (3). From the midgut lumen, the parasite traverses the peritrophic membrane and epithelial layer of the midgut and develops to oocysts (3). The oocysts invade other vector tissues such as salivary glands to complete the sporogonic development (3). In general, the susceptibility of mosquitoes to *Plasmodium* parasites infection is under genetic control (6–8), but the large variability in oocyst number among closely related mosquitoes indicates that environmental factors also play a role. Of special interest are the interactions between the vector, its microbiota and transmitted pathogens, since commensal bacteria interact with mosquito-borne pathogens (9) and can facilitate (10) or compete (11) with pathogen colonization and development within the vector midguts, prompting research into microbiota manipulation and transmission-blocking strategies (12). Depleting vector microbiota from bacteria that facilitates pathogen development could be exploited as a mean for blocking transmission. For example, the bacterium *Asaia bogorensis* increases midgut pH promoting *Plasmodium berghei* gametogenesis within *Anopheles stephensi* (10), and high abundance of Enterobacteriaceae increases *Plasmodium falciparum* infection in *Anopheles gambiae* midgut (13), making the reduction of these bacterial species a sound strategy to reduce pathogen infection in the vector and potentially block transmission to the host.

Although targeting specific commensal bacteria could block pathogen transmission, the lack of tools for the precise manipulation of the vector microbiota is currently a major limitation to developing novel transmission-blocking strategies. Specific host antibodies (Abs) are easily induced by vaccination, and once taken with the blood meal, they remain functional within the vector tissues and can bind symbionts (14) and other bacterial microbiota (15) within hematophagous arthropods (16). Surprisingly, host Abs specific to bacterial microbiota had never been used for microbiota manipulation and transmission blocking strategies (16). It was recently shown that anti-microbiota vaccines modulate the tick microbiota in a taxon-specific manner (17). Firstly, combining next-generation sequencing (NGS) and network analysis, Enterobacteriaceae was identified as a keystone taxon (i.e., highly connected taxa driving community composition and function) in the microbiota of *Ixodes ricinus* ticks (17, 18). Secondly, the abundance of vector-associated Enterobacteriaceae decreased in ticks fed on mice immunized with a live bacteria vaccine containing *Escherichia coli* (17). Thirdly, vaccination against Enterobacteriaceae had cascading ecological impacts on the whole tick microbiome by reducing bacterial diversity and modulating the functional profiles of the microbiome (17). Fourthly, decreased Enterobacteriaceae abundance was correlated with high levels of *E. coli*-specific Abs (17). Last but not least, no mortality or sign of pain were observed after the vaccination in the mice (17, 18) and the fecal microbiota of immunized mice showed no significant alterations (17). Here we tested whether modulation of mosquito microbiota by anti-microbiota vaccination of host birds against commensal Enterobacteriaceae disrupts *P. relictum* development within midguts and salivary glands of the vector *C. quinquefasciatus*.

## Results

### Anti-Microbiota Vaccination in Birds Interferes With Plasmodium-Induced Modulation of Mosquito Midgut Microbiota

**Figure 1:**
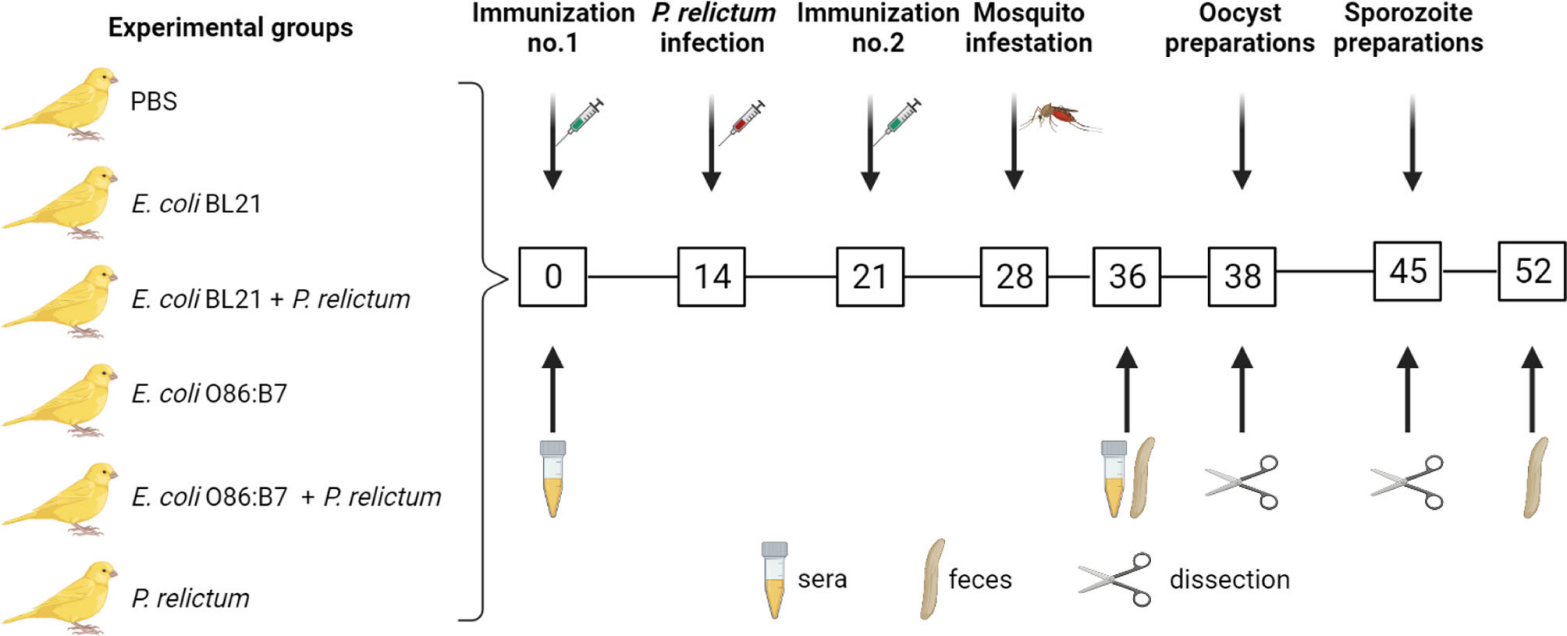
Experimental design and sample collection. Canaries were immunized twice (at day 0 and day 21) with a live vaccine containing *E. coli* BL21 (n = 7) or *E. coli* O86:B7 (n =7) or with a mock vaccine (PBS) (n = 3). A group of canaries (n = 5) and part of the birds vaccinated with *E. coli* BL21 (n = 4) or *E. coli* O86:B7 (n = 4) were infected with *P. relictum* at day 14. The birds were used as donors to feed *C. quinquefasciatus* (n = 20-50 mosquitoes per bird). The midgut of mosquitoes was dissected for microbiota (day 38 and day 45) and oocyst (day 38) analysis and salivary glands for sporozoite (day 45) analysis. Bird sera (used for ELISA and immunofluorescence) and fecal (used for bird gut microbiota analysis) samples were collected at different time points as indicated.

**Figure 2:**
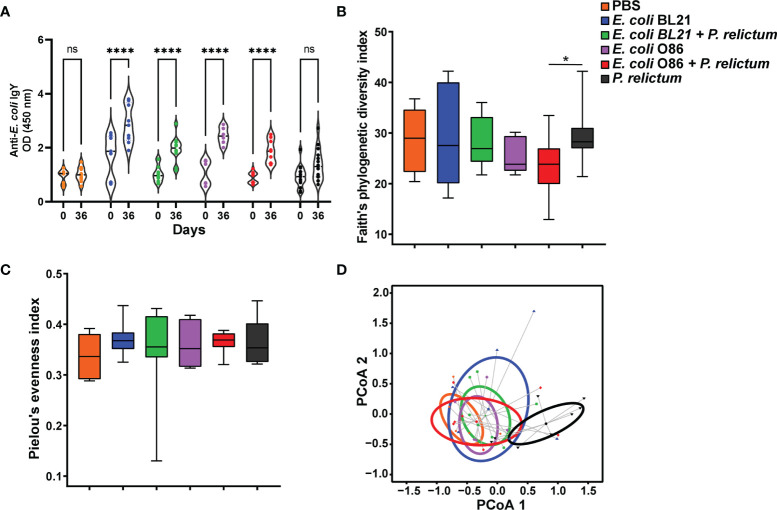
Impact of anti-microbiota vaccine and P. relictum infection on bird antibody response and mosquitoes’ microbial diversity **(A)**. Levels of IgY specific to *E. coli* proteins were measured by semiquantitative ELISA in sera of canaries before immunization (day 0) and in sera of canaries immunized with a mock vaccine, *E. coli* BL21 and E. coli O86:B7 alone or challenged with *P. relictum* (day 36 after first vaccination) (****p < 0.00001; ns, not significant). **(B)** Faith’s phylogenetic diversity and **(C)** Pielou’s evenness indexes were used to measure the richness and evenness, respectively, of microbiota of mosquitoes fed on canaries immunized with a mock vaccine, *E. coli* BL21 and *E. coli* O86:B7 alone or challenged with *P. relictum*. The asterisk indicates significative differences between groups (pairwise Kruskal-wallis, *p* = 0.03). **(D)** Beta diversity of mosquito microbiota in the different experimental conditions represented in PCoA plot obtained by Betadisper function. There were no differences in the intragroup dispersions (variances) (ANOVA, *p* = 0.426), whereas differences were found in the community composition between groups (PERMANOVA, *p* = 0.001), specifically the group infected with *P. relictum* alone was different from the other groups. Taxonomic profiles at the level of ASVs, used to measure the alpha and beta diversity, were obtained from 16S rRNA sequences from midgut of mosquitoes fed on mock-immunized (n = 5 mosquitoes midgut pool), *E. coli* O86:B7-immunized (n = 5 mosquitoes midgut pool), *E. coli* O86:B7-immunized and challenged with *P. relictum* (n = 8 mosquitoes midgut pool) and challenged only with *P. relictum* (n = 8 mosquitoes midgut pool).

Immunization was followed by experimental malaria infection and mosquito infestation (**Figure 1**). Anti-microbiota vaccination of birds with *E. coli* O86:B7 or *E. coli* BL21 increased the levels of IgY specific to *E. coli* (**Figure 2A**). Anti-*E. coli* IgY also increased in the sera of birds that received both anti-microbiota vaccines and infection with *P. relictum* (**Figure 2A**). No significant change was observed in the levels of anti*-E. coli* Abs in birds that received the mock vaccination (PBS) or those only infected with *Plasmodium* (**Figure 2A**). Following the bacterial immunization and *P. relictum* infection in birds, *C. quinquefasciatus* mosquitoes were allowed to feed on the birds to acquire the parasites and/or anti*-E. coli* Abs. The midguts of fed mosquitoes were dissected and used for microbiota analysis.

Bacterial community composition and diversity were compared between groups to assess the impact of anti-microbiota vaccines and *P. relictum* infection on the mosquito midgut microbiota. Analysis of alpha diversity indexes showed that overall, the phylogenetic richness (**Figure 2B**) and evenness (**Figure 2C**) did not differ between experimental groups (Kruskal-Wallis, *p* > 0.05). However, pairwise comparisons between groups revealed a reduced taxonomic richness in mosquitoes from *E. coli* O86:B7 + *P. relictum* group compared to *P. relictum-*infected mosquitoes (Kruskal-Wallis, *p* = 0.036, **Figure 2B**). Beta diversity analysis revealed a significant difference in Bray Curtis dissimilarity index between the groups (PERMANOVA, F = 2.40, *p* = 0.001). Specifically, *Plasmodium*-infected mosquito microbiota shows a tendency to separate from the other groups. Furthermore, dispersion analysis of Bray Curtis dissimilarity index did not show significant differences between groups (BetaDisper F= 1.01, *p* = 0.426, **Figure 2D**).

**Figure 3:**
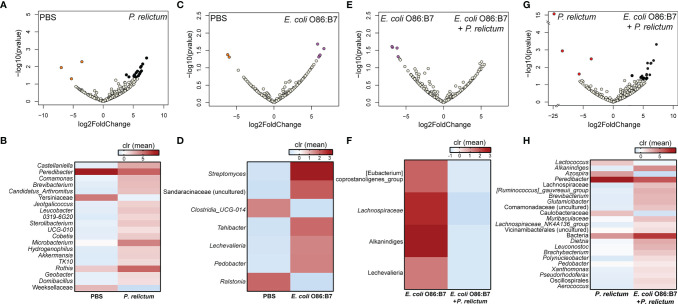
Impact of *E. coli* O86:B7 vaccine and *P. relictum* infection on the taxonomic profiles of mosquito microbiota. Volcano plot showing the differential bacterial abundance in mosquito microbiota between different experimental groups **(A)** PBS *vs. P. relictum*, **(C)** PBS *vs. E. coli* O86:B7, **(E)** *E. coli* O86:B7 *vs. E. coli* O86:B7+*P. relictum* and **(G)** *P. relictum vs*. *E. coli* O86:B7+*P. relictum*. Taxa with significant differences (Wald test, *p* < 0.05) between the groups are represented with colored dots (i.e., orange, purple, red and black). The gray dots represent taxa with no significant differences between groups. Heatmaps representing the relative abundance (expressed as clr) of taxa with significant differences between **(B)** PBS *vs. P. relictum*, **(D)** PBS *vs. E. coli* O86:B7, **(F)** *E. coli* O86:B7 *vs. E. coli* O86:B7+*P. relictum* and **(H)** *P. relictum vs*. *E. coli* O86:B7+*P. relictum*. Taxa with significant differences in their abundance were identified using DeSeq2 algorithm. The taxonomic profiles were obtained from 16S rRNA sequences from midgut of mosquitoes fed on mock-immunized (n = 5 mosquitoes midgut pool), *E. coli* O86:B7-immunized (n = 5 mosquitoes midgut pool), *E. coli* O86:B7-immunized and challenged with *P. relictum* (n = 8 mosquitoes midgut pool) and challenged only with *P. relictum* (n = 8 mosquitoes midgut pool).

**Figure 4:**
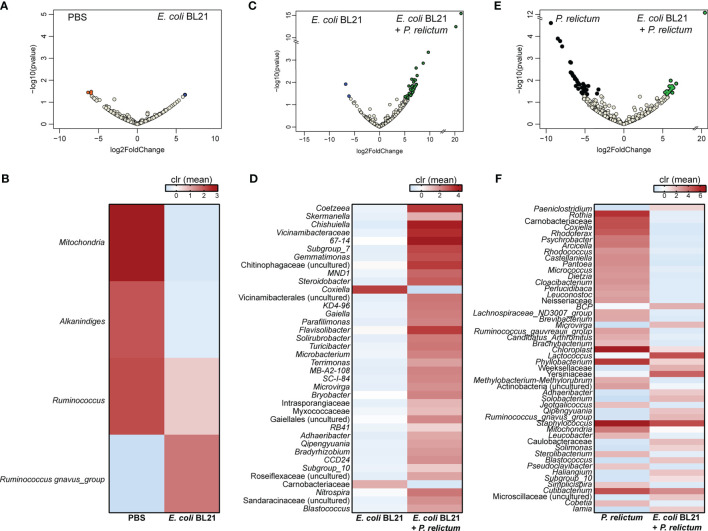
Impact of *E. coli* BL21 vaccine and *P. relictum* infection on the taxonomic profiles of mosquito microbiota. Volcano plot showing the differential bacterial abundance in microbiota of mosquitoes from different experimental groups **(A)** PBS *vs. E. coli* BL21, **(C)** *E. coli* BL21 *vs. E. coli* BL21+*P. relictum*, **(E)** *P. relictum vs*. *E. coli* BL21+*P. relictum*. Taxa with significant differences (Wald test, *p* < 0.05) between the groups are represented with colored dots (i.e., orange, blue, green and black). The gray dots represent taxa with no significant differences between groups. Heatmaps represent the relative abundance (expressed as clr) of taxa with significant differences in the comparisons **(B)** PBS *vs. E. coli* BL21, **(D)** *E. coli* BL21 *vs. E. coli* BL21+*P. relictum*, and **(F)** *P. relictum vs*. *E. coli* BL21+*P. relictum*. Taxa with significant differences in their abundance were identified using DeSeq2 algorithm. The taxonomic profiles were obtained from 16S rRNA sequences from midgut of mosquitoes fed on mock-immunized (n = 5 mosquitoes midgut pool), *E. coli* BL21-immunized (n = 7 mosquitoes midgut pool), *E. coli* BL21-immunized and challenged with *P. relictum* (n = 7 mosquitoes midgut pool) and challenged only with *P. relictum* (n = 8 mosquitoes midgut pool).

**Figure 5:**
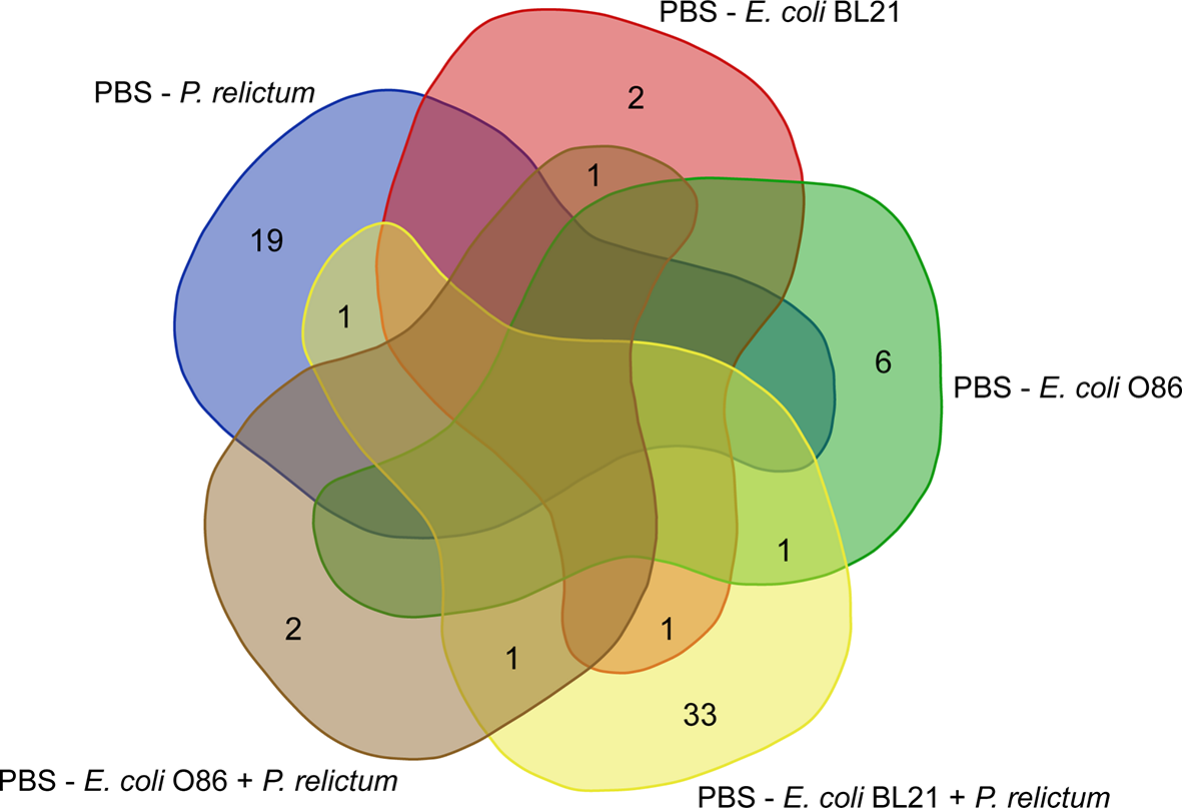
Comparison of unique and shared taxa across different experimental groups. Venn diagram showing the common and different taxa with significant changes in their abundance between vaccinated and infected groups, compared to the mock-vaccinated group (PBS).

Further characterization of the impact of anti-*E. coli* O86:B7 immunization and *Plasmodium* infection on mosquito midgut microbiota was achieved using differential abundance analysis. Significant changes in the abundance of 20 and 7 bacterial genera were detected in the midgut microbiota of mosquitoes fed on *P. relictum*-infected (**Figures 3A, B**) or *E. coli* O86:B7-immunized canaries (**Figures 3C, D**), respectively, compared to those fed on mock-immunized canaries. This suggests that *P. relictum* infection and the anti-microbiota vaccine disturb the mosquito microbiota. We next asked whether anti-microbiota vaccination could interfere with pathogen-induced modulation of the mosquito microbiota. The midgut microbiota of mosquito exposed to both *P. relictum* infection and anti-*E. coli* O86:B7 Abs had 23 and four taxa with significant changes in abundance compared to mosquitoes fed on *Plasmodium*-infected (**Figures 3E, F**), and *E. coli* O86:B7-immunized canaries (**Figures 3G, H**), respectively. Vaccination with *E. coli* BL21 was also associated with changes in the abundance of several bacterial taxa, compared with the control mock-vaccinated and with *Plasmodium* infection groups (**Figure 4**). Notably, compared with the mock-vaccinated group, the bacterial taxa affected by infection or vaccination alone were different between them and from those affected simultaneously by vaccination and infection (**Figure 5**). These results suggest that *P. relictum* infection and the anti-microbiota vaccines modulate the mosquito midgut microbiota in different ways and that the anti-microbiota vaccines interfere with the *Plasmodium*-induced modulation of the mosquito microbiota.

To rule out a negative effect of anti-microbiota vaccine on host microbiota, we collected bird feces after mosquitoes infestation (d36) and at the end of the experiment (d52). We compared fecal microbiota of mock-immunized, *E. coli* BL21-immunized and *E. coli* O86:B7-immunized birds. The results showed non-significant differences in the microbial richness (Kruskal-Wallis, *p* > 0.05), as measured by Faith’s phylogenetic index (**Supplementary Figure S1A**). Similarly, species evenness did not differ between the different experimental groups (Kruskal-Wallis, *p* > 0.05, **Supplementary Figure S1B**). Regarding the beta diversity of the microbial communities in bird guts, Bray Curtis dissimilarity index did not show significant differences among mock-immunized, *E. coli* BL21-immunized and *E. coli* O86:B7-immunized birds (PERMANOVA, F = 1.35, *p* = 0.25).

### Anti-Microbiota Vaccination Re-Structures the Microbial Communities in Midgut of Plasmodium-Infected Mosquitoes

**Figure 6:**
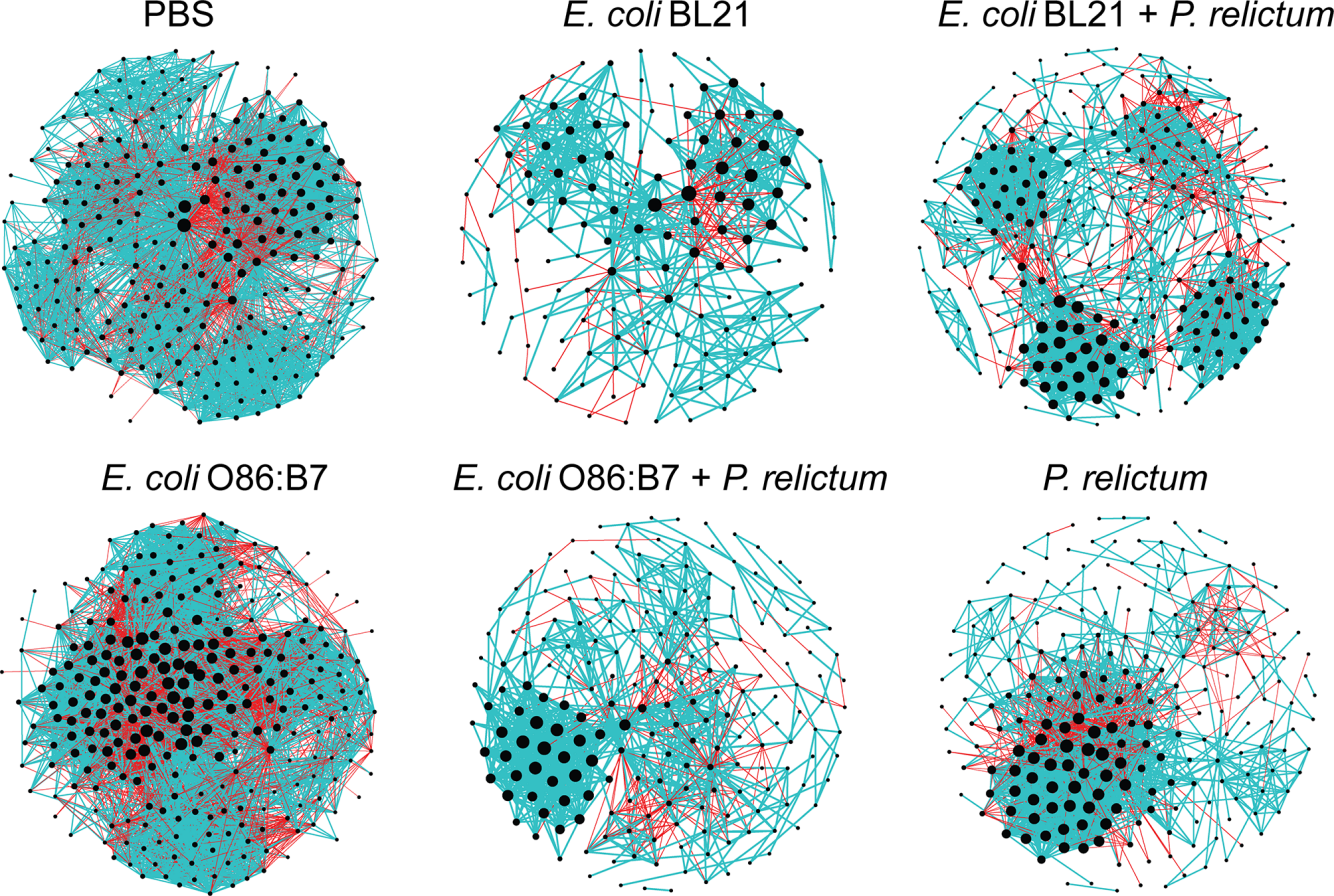
Co-occurrence networks of *C. quinquefasciatus* microbiota in the different experimental groups. Bacterial co-occurrence networks were inferred from the microbiota of mosquitoes fed on control (PBS), vaccinated and/or infected birds. Nodes represent bacterial taxa and connecting edges stand for a co-occurrence correlation (SparCC > 0.75). Node sizes are proportional to the eigenvector centrality value. Edges representing positive or negative correlations were colored in blue and red, respectively. Only nodes with at least one connection are displayed.

Bacteria co-occurrence networks were used to further characterize the impact of *Plasmodium* infection and anti-microbiota vaccines on the mosquito microbiota. Visual inspection of the taxonomic networks revealed that anti-microbiota vaccines and malaria infection, together and separately, changes network topology, compared with the control mock vaccine group (**Figure 6** and **Table 1**). Jaccard index was used to test for similarities (Jacc = 0, lowest similarity and Jacc = 1, highest similarity) in selected local network centrality measures (i.e., hub taxa, degree, betweenness centrality, closeness centrality, and eigenvector centrality) of the different networks. The observed Jaccard index for the betweenness centrality was higher than expected by random for the comparison between *E. coli* BL21 and *E. coli* O86:B7 networks (Jacc = 0,418, *p* = 0.01) (**Supplementary Table S1**). In addition, except for the Jaccard index of betweenness centrality in the PBS *- P. relictum* (Jacc = 0,432, *p* = 0.004), PBS - *E. coli* BL21 (Jacc = 0,512, *p* = 0.000002) and PBS - *E. coli* O86:B7 (Jacc = 0,522, *p* = 0. 000001) network comparisons, the observed Jaccard indexes of the other centrality measures were lower than expected by random in most networks (**Supplementary Table S1**). This suggests low similarity between compared networks. Topological differences, together with dissimilarity in local network centrality measures, indicate a major shift in the community structure induced by anti-microbiota vaccines and malaria infection.

**Table 1:** Topological parameters of co-occurrence networks.

Network features	PBS	*E. coli* BL21	*E. coli* BL21 + *P. relictum*	*E. coli* O86:B7	*E. coli* O86:B7 + *P. relictum*	*P. relictum*
Nodes	231	137	267	261	199	221
Edges	4417	591	1787	6629	991	1709
Positive	3,041 (68.9%)	476 (80.5%)	1,386 (77.6%)	3,827 (57.7%)	822 (82.9%)	1,232 (72.1%)
Negative	1,376 (31.2%)	115 (19.5%)	401 (22.4%)	2,802 (42.3%)	169 (17.1%)	477 (27.9%)
Network diameter	3	10	13	4	10	12
Average degree	38.242	8.628	13.386	50.797	9.96	15.466
Weighted degree	12.07	4.265	6.113	7.018	5.287	5.83
Average path length	1.909	3.47	3.871	1.993	3.609	3.716
Modularity	1.459	0.974	1.105	2.996	0.785	0.872
Number of modules	6	18	27	13	20	32
Average clustering coefficient	0.719	0.556	0.621	0.658	0.538	0.623

**Figure 7:**
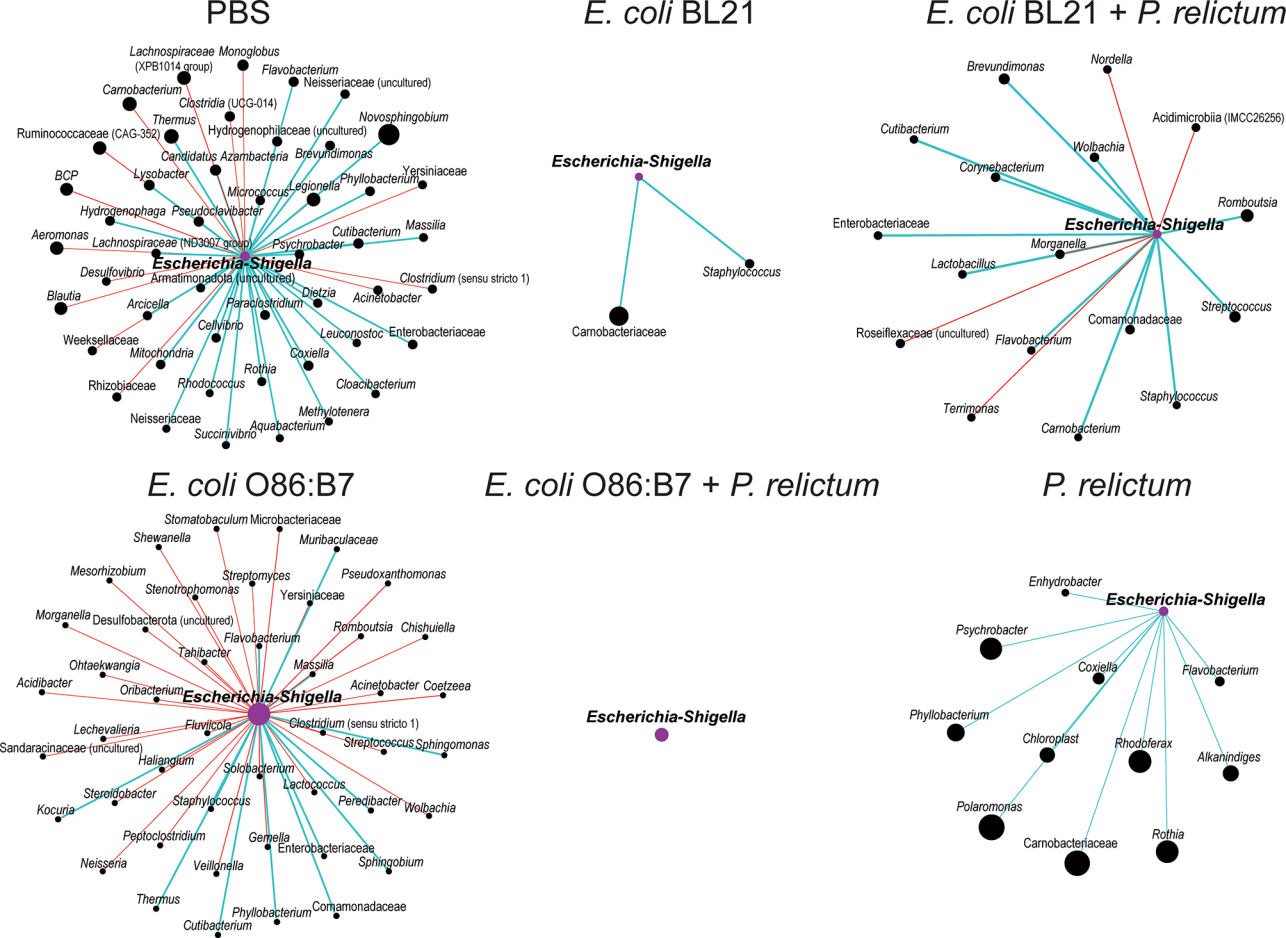
Local connectivity of *Escherichia-Shigella* in the co-occurrence networks of *C. quinquefasciatus* microbiota in the different experimental groups. The nodes/taxa linked to *Escherichia*-*Shigella* (purple node) were identified in the bacterial co-occurrence networks of mosquitoes fed on mock-immunized (PBS), *E. coli*-immunized and infected birds. Node sizes are proportional to the eigenvector centrality value. Only nodes with at least one connection are displayed. Connecting edges stand for a co-occurrence correlation (SparCC > 0.75), representing positive (blue edges) and negative (red edges) interactions between bacteria. BCP – *Burkholderia Caballeronia Paraburkholderia*.

High variation of local connectedness of *Escherichia-Shigella* was observed in co-occurrence sub-networks of experimental groups (**Figure 7**). Specifically, the number of direct neighbors co-occurring with *Escherichia-Shigella* in the microbiota of mosquitoes fed on birds of the different experimental groups decreased in comparison to the sub-network of the microbiota of mosquitoes fed on mock-vaccinated birds. Although the number of edges directly connected to *Escherichia-Shigella* was similar in the sub-networks of the microbiota of mosquitoes fed on mock-immunized (47 co-occurring taxa) and *E. coli* O86:B7-immunized birds (45 co-occurring taxa), an increase of negative co-occurrence correlation was observed in the latter compared to the former group. A detailed analysis of the nodes connected to *Escherichia-Shigella* revealed the inexistence of shared taxa among the different sub-networks (**Supplementary Figure S2**). In addition, we used the eigenvector centrality metric to evaluate the “keystoneness” (importance of a node within the network) of *Escherichia-Shigella* in the different microbial networks. Eigenvector centrality value of *Escherichia-Shigella* in the sub-network of mosquitoes fed on *E. coli* BL21-immunized (eigenvector 0.03) and *E. coli* O86:B7-immunized birds (eigenvector 1) decreased and increased, respectively, compared to that in the control sub-network (i.e., mock vaccine group, eigenvector 0.29). Interestingly, eigenvector centrality values of *Escherichia-Shigella* decreased dramatically in the groups *E. coli* BL21+*P. relictum* (eigenvector 0.07), *E. coli* O86:B7+*P. relictum* (eigenvector 0), compared with the *P. relictum* infected mosquito group (eigenvector 0.16). Variable local interactions and “keystoneness” of *Escherichia-Shigella* between networks suggest that this taxon is affected by anti-microbiota vaccines and *Plasmodium* infection. More importantly, *Escherichia-Shigella* losses importance in the microbiota of mosquito exposed simultaneously to anti-*E. coli* Abs and *P. relictum*.

### Anti-Microbiota Vaccination Reduces Plasmodium Infection Within Mosquito Tissues

**Figure 8:**
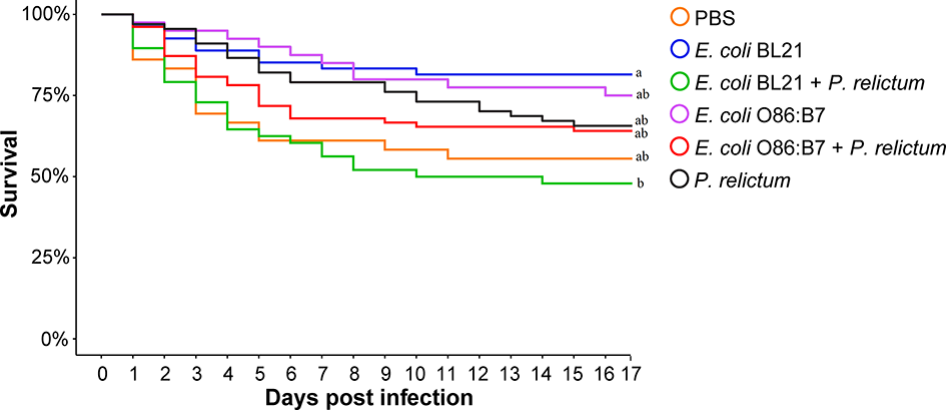
Survival of blood-fed mosquitoes throughout the experiment. The highest mortality was observed in the mosquito group fed on donors vaccinated with *E. coli* BL21 and infected with *P. relictum*, which significantly differed from the group fed on the birds vaccinated with *E. coli* BL21 (*p* = 0.02). Survival of mosquitoes between other groups did not differ significantly. Survival of mosquitoes was estimated by Fisher’s exact test with Bonferroni correction. Different letters at the end of the curves indicate statistically significant differences in survival rate (*p* < 0.05).

Mosquito survival was recorded and compared between groups. Results showed a significant difference in survival rate between the groups (Fisher’s exact test, *p* = 0.013). However, pairwise comparison of individual groups revealed significant differences only in the survival of mosquitoes fed on birds of the *E. coli* BL21 and *E. coli* BL21-*P. relictum* groups between them (*p* = 0.02) and with the others (**Figure 8**). Mosquitoes in the *E. coli* BL21 and *E. coli* BL21+*P. relictum* groups had the highest and lowest survival rates.

**Figure 9:**
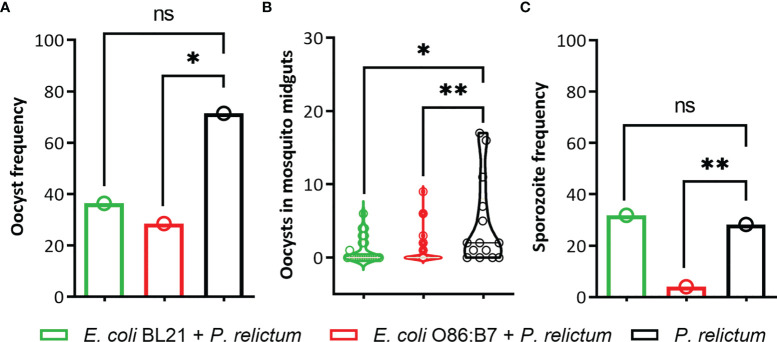
Impact of anti-microbiota vaccines on P. relictum development in infected mosquitoes. Three groups of P. relictum-infected birds were used as infection donors for mosquitoes. One of the groups was vaccinated with *E. coli* BL21, the other with *E. coli* O86:B7, and a third group was a control (only infection). **(A)** The percentage of mosquitoes in which at least one oocyst was found in the midgut by microscopy and compared between the groups was measured. **(B)** The number of oocysts in the midgut of P. relictum-infected mosquitoes was calculated and compared between the groups. **(C)** The percentage of mosquitoes in which sporozoites were detected in salivary glands by microscopy or PCR was measured and compared between the groups. The parameters were compared between groups by Fisher’s exact test with Bonferroni correction (oocyst and sporozoite frequency) and the Mann-Whitney U test (number of oocyst). (*p <0.05; **p <0.001; ns, not significant; n = 14-53 mosquitoes per group).

The occurrence (i.e., number of mosquitoes in which at least one oocyst was found) and number of *P. relictum* oocysts in midguts and occurrence of sporozoites in salivary glands of mosquitoes were measured. The results showed a significant overall difference in the occurrence of oocysts between the groups (Fisher’s exact test, *p* = 0.03). Pairwise comparisons revealed that the occurrence of *Plasmodium* oocysts was significantly lower in the midguts of mosquitoes fed on birds vaccinated with *E. coli* O86:B7 (*p* = 0.01), but not with *E. coli* BL21 (*p* > 0.05), compared with mosquitoes infected with *P. relictum* and not exposed to anti-*E. coli* Abs (**Figure 9A**). The number of oocysts was significantly lower in midguts of mosquitoes fed on birds vaccinated with both *E. coli* O86:B7 (*p* = 0.01) and *E. coli* BL21 (*p* = 0.001), compared with control mosquitoes (**Figure 9B**). We then hypothesized that decreased oocysts load in midguts might be associated with lower sporozoite infection in salivary glands. Results showed a significant difference between sporozoite occurrence between the groups (Fisher’s exact test, *p* < 0.001). The occurrence of sporozoites in salivary glands of mosquitoes fed on *E. coli* O86:B7-immunized, but not *E. coli* BL21-immunized (*p* > 0.05), birds was significantly lower (*p* = 0.001) than the control group (i.e., *P. relictum* infection alone) (**Figure 9C**).

## Discussion

In this study, we showed that anti-microbiota vaccination of birds with two strains of *E. coli* O86:B7 and BL21 modulate *C. quinquefasciatus* midgut microbiota. These results concur with a previous report in which live bacteria vaccine were used as a tool for the manipulation of tick microbiome (17, 18). During feeding, hematophagous ectoparasites ingest blood from the vertebrate host, along with host immune molecules. Host Abs and/or complement proteins have been detected in the guts of ticks (19–24), mosquitoes (25, 26), sandflies (27, 28), and tsetse flies (29). Once ingested, host immune components can remain active from a few hours to months depending on the species of blood-sucking arthropod, raising the possibility that vertebrate Abs could interact with pathogens and microbiota (16). Empirical work shows that host Abs can target vector-borne pathogens within ticks (30) and mosquitoes (31–33). Targeting pathogen proteins expressed within the arthropod vectors is the rationale behind transmission-blocking vaccines (32–34). Functional host Abs have also been shown to interact with symbionts in *Rhodnius prolixus* (14) and *Glossina morsitans* (35), as well as with bacterial microbiota in mosquitoes (15) and ticks (17, 18). Vaccination of mice with *E. coli* BL21 induced anti-*E. coli* Abs that when ingested with the blood decreased Enterobacteriaceae abundance within the tick microbiota (17). Targeting commensal *Escherichia* in ticks with anti-*E. coli* Abs reduced the connectivity of this taxon in the co-occurring networks (17). Similarly, in this study we observed reduced connectivity of *Escherichia* in the co-occurring networks of mosquitoes fed on birds vaccinated with *E. coli* BL21, but not in those vaccinated against *E. coli* O86:B7. However, we did observe a switch in the bacterial connectivity pattern of mosquitoes fed on *E. coli* O86:B7 compared to the control group PBS. In the control group, the majority of *Escherichia* correlations with its direct neighbor nodes were positive, while in the *E. coli* O86:B7 group most correlations were negative. These results show that antibody-mediated disturbance of the microbiome had cascading ecological impacts on the whole tick microbiome with strong impact on the structure of the microbial community of the vectors.

Recent research on vector-pathogen-microbiota interactions shows that microbial communities within vectors strongly influence pathogen colonization and transmission (36). Despite recent advances in vector microbiota research, the lack of tools for the precise and selective manipulation of the vector microbiome is currently a major limitation to disrupt pathogen-microbiome interactions in a taxon-specific manner (12, 37). Here we showed that mosquitoes fed on birds immunized with the Enterobacteriaceae bacteria *E. coli* O86:B7 and *E. coli* BL21 had reduced numbers of *P. relictum* oocysts in the midguts. The results support the potential role of commensal Enterobacteriaceae as a key player in the early development of *Plasmodium* within the vector. In agreement with this, high abundance of Enterobacteriaceae was previously associated with increase *P. falciparum* infection in *A. gambiae* midgut (13), and several studies showed that the ookinete–oocyst transition of *Plasmodium* spp. is strongly influenced by resident midgut microbiota in different mosquito species (38–41). However, the directionality (facilitation *vs.* competition) of midgut microbiota contribution to infection cannot be simplified to the abundance of a single taxon, while neglecting global changes of the microbial community as a whole. For example, experimental exposure of mosquitoes to a cocktail of penicillin and streptomycin (PS) reduced the proportion of microbiota-resident Enterobacteriaceae 92-fold, while simultaneous exposure to PS and *Plasmodium* infection increased the prevalence and intensity of *P. berghei* oocyst in *A. gambiae* midguts (42). This is not expected if Enterobacteriaceae facilitates *Plasmodium* infection (13). However, regardless that the impact of Enterobacteriaceae on parasite infectivity may differ between *Plasmodium* species (e.g., *P. berghei vs*. *P. falciparum*), the decreased of Enterobacteriaceae has been associated with a 19-fold increase of *Asaia* sp. in PS-exposed *A. gambiae* (42). Commensal *Asaia bogorensis* remodels glucose metabolism and increases midgut pH which in turn induces *P. berghei* gametogenesis and facilitates parasite infection of *A. stephensi* (10). Thus, the decrease of Enterobacteriaceae abundance may be balanced by the increase of another taxon (e.g., *Asaia* sp.) that also facilitates infection with a net effect of increased *Plasmodium* infectivity. Our results showed that anti-microbiota vaccines altered the *Plasmodium*-induced modulation of the mosquito microbiota, resulting in a global microbial community transformation and the reduction of *Plasmodium* infectivity.

We also found a significant reduction of sporozoites occurrence in the salivary glands of mosquitoes fed on *E. coli* O86:B7-immunized birds, but no in those fed on *E. coli* BL21-immunized birds. This could be explained by differences in the levels of the glycan Galα1-3Gal (α-Gal) in these two *E. coli* strains. The strain *E. coli* O86:B7 expresses high levels of α-Gal (43), which is not the case for *E. coli* BL21 (44, 45). Even when *E. coli* BL21 produces low levels of α-Gal, immunization with this bacterium induces anti-α-Gal (18), as low-abundant antigens could also induce immune responses. However, immunization with *E. coli* O86:B7 may induce anti-α-Gal Abs at levels even higher than *E. coli* BL21. This is relevant because as previously shown in ticks (18), α-1,3-galactosyltransferase genes, and possibly α-Gal, may be widely distributed in mosquito bacterial microbiota, increasing the number of bacterial taxa targeted by Abs induced by *E. coli*-vaccination. Another point to consider is that α-Gal has been detected on the surface of *P. falciparum*, *P. berghei* and *Plasmodium yoelii* sporozoites (43). Whether *P. relictum* sporozoites express α-Gal within *C. quinquefasciatus* salivary glands and whether anti-α-Gal Abs could target this *Plasmodium* stage within mosquito salivary glands remain to be tested.

Interestingly, mosquitoes fed on birds immunized with *E. coli* BL21 or *E. coli* O86:B7 had the highest survival rates, although the differences were significant only for mosquitoes in the *E. coli* BL21 group, compared with the *P. relictum*+*E. coli* BL21 group. Similar impact on survival was observed on mosquitoes fed on blood supplemented with PS (42). Exposure of *A. gambiae* to PS increased survival and fecundity, which are known to augment vectoral capacity (42). Our results can be interpreted as if microbiota modulation by *E. coli* BL21 vaccination: (i) increases *C. quinquefasciatus* fitness, and/or as if microbiota modulation by *E. coli* BL21 vaccination together with *Plasmodium* infection (ii) increases *P. relictum* virulence on mosquitoes and/or (iii) induces changes in the microbiome that impose a high cost on mosquito midguts homeostasis.

## Conclusions

The vector microbiome can be assembled in different possible states, some of which may be incompatible with pathogen infection and/or transmission, while others increase vector competence or could increase or reduce vector fitness. Unraveling how to modulate these different states in a precise manner offers a powerful tool to develop novel transmission-blocking vaccines (16). Our results support the use of anti-microbiota vaccines to target vector commensal bacteria that facilitate pathogen infection (16, 46). In addition to taxon-specific effects, the community-level effects and cascading ecological impact of anti-microbiota vaccines on vector microbiota might induce infection-refractory states in the vector microbiome. Effective infection by vector-borne pathogens involves competent vectors, infective pathogens, and an infection-compatible microbiome (16). Mismatch of at least one of these components can result in an impaired ability of the vector to support the pathogen life cycle. For example, one strategy used to reduce the vector competence for pathogens is the genetic modification of insects that no longer transmit pathogens (47). Our results provide strong evidence that alterations in the vector midgut microbiomes, without the need to altering vector and/or pathogen genetics, affect pathogen infection in the vector. Therefore, deviations from infection-compatible microbiomes could block transmission and disease development. Anti-microbiota vaccines can be used as a microbiome manipulation tool for the induction of infection-refractory states in the vector microbiome for the control of major vector-borne pathogens such as malaria.

## Material and Methods

### Ethical Statement

All procedures were performed at the Nature Research Centre in Vilnius, Lithuania, according to Lithuanian and International Guiding Principles for Biomedical Research Involving Animals (2012). Infection experiments and other procedures were reviewed and approved by the Lithuanian State Food and Veterinary Service, Ref. No. 2020/07/24-G2-84. The assessment of the animal health and all described procedures were implemented by trained professionals (under licenses 2012/02/06-No-208, and 2016/01.29-No-344).

### Birds and Housing Conditions

Seven-months-old domestic canaries were purchased commercially and kept for adaptation for one-month before experimental procedures. Birds were kept at the Nature Research Centre vivarium (License No. LT-61-13-003) under standard living conditions for birds. Experimental animals were housed in cages, up to three birds per cage. The facilities were under a controlled temperature of 21°C, which was maintained throughout the time that the experimentation lasted. Animals were supplied with a standard food for canaries and water *ad libitum*.

### Experimental Design

Domestic canaries (*Serinus canaria domestica*) were used as a model for avian malaria infection, anti-microbiota vaccination and as donors of *P. relictum* to mosquitoes. Before the experimental procedures began, birds were randomly separated into six groups as displayed in **Figure 1**. Birds in one group received a mock vaccination containing PBS and adjuvant. Another group of birds was only infected with *P. relictum*. All birds in the other four groups received anti-microbiota vaccines and in two of these groups, birds were additionally infected with *P. relictum*. After *Plasmodium* infection and antibody titers to anti-microbiota vaccines were confirmed, *C. quinquefasciatus* mosquito females were fed on the birds for pathogen acquisition experiment. In total, 22 experimental birds from all groups were exposed to mosquito bites as described in the sections below. Engorged mosquitoes (n = 663) were used for midgut and/or salivary glands preparations. Mosquito tissues were used for oocyst (midguts) and sporoites (salivary glands) counting. Mosquito midguts were also used for DNA extraction and microbiota analysis using bacterial 16S rRNA amplicon sequencing. Bird feces were collected to test for an impact of anti-microbiota vaccines on bird gut microbiota. Experimental procedures are described below.

### Bacterial Cultures and Live Bacteria Immunization

Representative bacteria of the genus *Escherichia*-*Shigella* were selected to be included in live bacteria vaccine formulations as previously reported (17, 18). Anti-microbiota vaccinations were used to test the impact of host immune response against targeted bacteria on mosquito microbiota composition and structure, mosquito survival and *Plasmodium* infection. The *Escherichia coli* strains BL21 (DE3, Invitrogen, Carlsbad, CA, USA), and O86:B7 (ATCC^®^ 12701TM) were selected. The two bacterial strains were prepared as previously described (17, 18). Briefly, *E. coli* was grown on Luria Broth (LB, Sigma-Aldrich, St. Louis, MO, USA) at 37°C under vigorous agitation, washed with phosphate buffer saline (PBS) 10 mM NaH2PO4, 2.68 mM KCl, 140 mM NaCl, pH 7.2 (Thermo Scientific, Waltham, MA, USA), resuspended at 3.6 × 10^4^ colony-forming unit (CFU)/mL, and homogenized using a glass homogenizer. Eight-month-old, canaries were immunized subcutaneously with *Escherichia* sp. in 50 µL (4, 1 × 10^6^ CFU per bird) of a water-in-oil emulsion containing 70% (w/w) Montanide™ ISA 71 VG adjuvant (Seppic, Paris, France), with a booster dose two weeks after the first dose. Control birds received a mock vaccine containing PBS and adjuvant. All reagents used for bacterial preparation were apyrogenic.

### Bacterial Protein Extraction

*Escherichia coli* strains were washed twice with PBS, centrifuged at 1,000× g for 5 min at 4°C, resuspended in 1% Trion-PBS lysis buffer (Sigma-Aldrich, St. Louis, MO, USA) and homogenized with 20 strokes using a glass balls homogenizer. The homogenate was then centrifuged at 300× g for 5 min at 4°C and the supernatant was collected. Protein concentration was determined using the Bradford Protein Assay (Thermo Scientific, San Jose, CA, USA) with Bovine Serum Albumin (BSA) as standard. Bacterial protein extracts were used in the indirect ELISA to measure anti-*E. coli* Abs in bird sera.

### Thawing the Cryopreserved Avian Malaria Sample

The cryopreserved *P. relictum* strain SGS1 was thawed and used to infect donor canaries. Tubes containing *Plasmodium*-infected avian blood, conserved frozen in liquid nitrogen, were thawed as described by Dimitrov et al. (48). Briefly, thawed samples were mixed with 12% NaCl (1/3 of thawed sample amount). After 5 min equilibration, one volume of 1.6% of NaCl was added followed by centrifugation at 10,000 rpm for 5 min. After centrifugation, the supernatant was removed and 1.6% NaCl (1/3 of original sample) was added and centrifuged again. The same procedure was repeated three times with 0.9% NaCl solution. The final mixture was diluted with 0.9% NaCl and sub-inoculated into two canaries.

### Experimental Infection of Birds

All birds from the mock-immunized group and birds randomly selected (n = 4) from *E. coli* BL21-immunized and *E. coli* O86:B7-immunized groups were experimentally infected with *P. relictum* (SGS1) using the protocol described by Palinauskas et al. (49). Each experimental bird was sub-inoculated with a mixture (0.10 mL) of infected blood, 3.7% sodium citrate and 0.9% saline in proportion 4:1:5 into the pectoral muscles. Each bird received approximately 1 x 10^5^ of mature *P. relictum* meronts. The duration of experimental time before exposure to the mosquitoes was 13 days post inoculation (dpi). This time was sufficient to develop higher parasitemia in the blood for malaria parasites. Birds with suitable infection levels were exposed to feed mosquitoes. Parasitemia was examined every 4 days by taking blood from the brachial vein as described in the section below.

### Maintenance of Mosquitoes

For experimental infection of *P. relictum* in mosquitoes, we used the P. B. Šivickis parasitology laboratory-reared *C. quinquefasciatus* mosquitoes. The colony was maintained as described in Žiegytė et al. (50). Mosquitoes were kept in a nylon netted cage (65×65×65 cm) under controlled conditions (room temperature 23 ± 1°C; humidity 75-80%; photoperiod 17:7 light:dark). Adult insects were provided with cotton wools saturated with 5% saccharose solution. Mosquito females were randomly separated from the main colony into smaller cages (about 300 mosquitoes) for each experimental group. For experimental infection we used insects of the same age, approximately one week after hatching.

### Mosquito Exposure to Vaccinated Birds and Infection With Plasmodium

We evaluated gametocytemia in all donor birds immediately after mosquito exposure. The gametocytemia of *P. relictum*-infected donor birds varied between 0.005% – 1.5%, *E. coli* BL21-immunized birds – 0.02% – 2% and *E. coli* O86:B7-immunized birds – 0.01% – 3.5%. For experimental exposure to mosquito bites, the donor bird was carefully immobilized and fixed in a paper tube, leaving only the legs exposed for the mosquitoes (51). The tube was placed into a separate mosquito cage with separated female mosquitoes taken from the main colony. The bird was kept up to one hour, or when approximately 40 fully saturated mosquitoes were counted. Engorged insects were separated into small cages (17.4 × 17.5 × 17.5 cm) and kept there up to 17 days post exposure (dpe) under the same rearing conditions as described above. All cages were additionally provided with cups containing water for oviposition. Mosquitoes were exposed to donor birds immunized with *E. coli* BL21, *E. coli* O86:B7 and mock vaccine 28 days post first vaccination (dpvI). *Plasmodium-*infected donor birds were used for mosquito infestation 28 dpvI (i.e., 14 dpi). Exposed mosquitoes were dissected gradually for preparations of different sporogonic stages and sampling for microbiota analysis.

### Blood Sample and Bird Feces Collection

Blood samples were taken from birds by puncturing brachial vein using microcapillaries. A small drop of blood was used to make smears for microscopy to estimate the development of parasites in the blood. Smears were air-dried, fixed with absolute methanol and stained as described by Valkiūnas et al. (52). A fraction of blood (20-30 μL) was placed in SET-buffer for molecular analysis (PCR, see below) to confirm the lineage in recipient birds. The rest of the blood (100 μL) was used to obtain serum for immunological analysis. Before centrifugation the blood was incubated for 2 h at room temperature, allowing it to coagulate. Then samples were centrifuged at 5,000x g for 5 min and serum separated in microtube and kept in a freezer at -15°C until processing. The blood for smears and SET-buffer was collected on days 0, 4, 8, 12, after inoculation of parasites. The blood for serum was taken on days 0 and 36 after the first vaccine inoculation. Fresh feces were collected from each bird in sterile tubes on days 36 and 52 and were stored at -20°C before genomic DNA extraction.

### Microscopic Examination

We used an Olympus BX61 light microscope (Olympus, Japan) to examine blood smears and preparations of mosquito tissues. Parasitemia was calculated as a percentage by actual counting of the number of parasites per 10,000 erythrocytes as described by Godfrey et al. (53). The infection intensity in the mosquito was evaluated by counting the oocysts in the midgut. Successful sporogony was determined by examining salivary glands preparations and confirming sporozoite development.

### Indirect ELISA

The levels of Abs reactive to bacterial proteins were measured in bird sera as previously reported (17, 18), with small modifications. The 96-well ELISA plates (Thermo Scientific, Waltham, MA, USA) were coated with 50 ng/mL (100 µL/well) of *E. coli* BL21 protein extracts in carbonate/bicarbonate buffer (0.05 M, pH 9.6) and incubated for 2 h with 100 rpm shaking at RT. Subsequently, plates were incubated overnight at 4°C. Wells were washed three times with 100 µL of PBS containing 0.05% (vol/vol) Tween 20 (PBST), and then blocked by adding 100 µL of 1% Human Serum Albumin (HSA)/PBS for 1 h at RT and 100 rpm shaking. After three washes, sera samples, diluted at 1:200 in 0.5% HSA/PBS, were added to the wells and incubated for 1 h at 37°C and 100 rpm shaking. The plates were washed three times and HRP-conjugated Abs (goat anti-turkey IgG) (MyBioSource, San Diego, CA, USA) were added at 1:1,000 dilution in 0.5% HSA/PBST (100 µL/well) and incubated for 1 h at RT with shaking. The plates were washed three times and the reaction was developed with 100 µL ready-to-use TMB solution (Promega, Madison, WI, USA) at RT for 20 min in the dark, and then stopped with 50 µL of 4% H_2_SO_4_. Optimal antigen concentration and dilutions of sera and conjugate were defined using titration assays. The optical density (OD) was measured at 450 nm using an ELISA plate reader (Filter-Max F5, Molecular Devices, San Jose, CA, USA). All samples were tested in triplicate and the average value of three blanks (no Abs) was subtracted from the reads. The cut-off was determined as two times the mean OD value of the blank controls.

### Evaluation of Mosquito Survival and Collection of Midguts and Salivary Glands for Testing *P. relictum* Development and Midgut Microbiota Analyses

The survival of mosquitoes was estimated by daily checking the mosquito cages at 10 am and counting dead insects until 17 dpe. On 10 dpe, mosquito midguts were dissected for estimation of developed oocysts and microbiota analysis. Before dissection, mosquitoes were euthanized by shaking vigorously to stun them in insect aspirator. Wings and legs of the insects were removed before dissection, which was performed under the binocular stereoscopic microscope. Each mosquito was carefully separated in two segments, the thorax with head and abdomen. The abdomen was placed in the drop of saline and the midgut of the mosquito was extracted. The midgut was stained according to Kazlauskienė et al. (51) for counting oocysts of *Plasmodium* parasite. For microbiota analysis, unstained midguts were pooled up to 10 in sterile microtubes and frozen at -20C. To eliminate contamination of samples, new dissecting needles were used for each dissected insect. On 17 dpe, each mosquito was carefully separated in two segments, the thorax with head and abdomen. Salivary glands were extracted from the thorax, placed in a separate sterile drop of saline and grinded to make a smear to record the presence of sporozoites (51). The remnants of salivary gland preparations and thorax were fixed in SET-buffer for PCR analysis. The abdomen, the same as on day 10, was placed in a drop of saline to prepare the samples for the midgut microbiota analysis.

### DNA Extraction and PCR for *P. relictum* Identification

Total DNA for PCR analysis was extracted from the blood remnants of mosquitoes using an ammonium acetate extraction protocol by Sambrook & Russel (54). A nested PCR protocol described by Hellgren et al. (55) was used to confirm *P. relictum* infection in the donor birds and test mosquitoes for positive parasite sporozoite development. For the first PCR we used the primers HaemNFI [5’-CATATATTAAGAGAAITATGGAG-3’] and HaemNR3 [5’-ATAGAAAGATAAGAAATACCATTC-3’] (55). In the second PCR a mitochondrial *cyt b* gene (478 bp) was amplified using the primers HaemF [5’-ATGGTGCTTTCGATATATGCATG-3’] and HaemR2 [5’-GCATTATCTGGATGTGATAATGGT-3’] (56). For PCR mix we used 12.5 μl of DreamTaq Master Mix (Thermo Fisher Scientific, Lithuania), 8.5 μl of nuclease-free water, 1 μl of each primer and 2 μl of template DNA (extracted DNA or products of first PCR). *P. relictum*-positive samples were determined by running 2 μl of second PCR product on 2% agarose gel. For parasite lineage confirmation, samples containing parasite DNA were sequenced from the 5` end using the HAEMF primer on an ABI PRISM TM 3100 capillary sequencing robot (Applied Biosystems, USA) as described by Bensch et al. (56). The BLAST search tool (National Centre for Biotechnology Information website: http://www.ncbi.nlm.nih.gov/BLAST) was used to determine SGS1 lineage.

### DNA Extraction and 16S rRNA Sequencing for Microbiota Analysis

Genomic DNA for microbiota analysis was extracted from frozen midguts of engorged mosquitoes and from fecal samples of birds using a Pure Link Microbiome DNA Purification Kit (Invitrogen, Thermo Fisher Scientific, CA, USA). Each DNA sample was eluted in 100 µl of elution buffer. Genomic DNA quality (OD260/280 between 1.8 –2.0) was measured with NanoDrop™ One (Thermo Scientific, Waltham, MA, USA). More than 850ng of DNA at ≥ 8.5 ng/μL concentration were sent for amplicon sequencing of the bacterial 16S rRNA gene, which was commissioned to Novogene Bioinformatics Technology Co. (London, UK). Libraries were prepared with NEBNext^®^ Ultra™ IIDNA Library Prep Kit (New England Biolabs, MA, USA). A single lane of Illumina MiSeq system was used to generate 251-base paired-end reads from the V4 variable region of the 16S rRNA gene using barcoded universal primers (515F/806R) in samples from mosquitoes engorged on *E. coli* BL21-immunized (n = 7 midgut pools), *E. coli* BL21-immunized and *Plasmodium*-infected (n = 7), *E. coli* O86:B7-immunized (n = 5), *E. coli* O86:B7-immunized and *Plasmodium*-infected (n = 8), *Plasmodium*-infected (n = 8) or mock-immunized (n = 5) birds. The raw 16S rRNA sequences obtained from mosquito samples were deposited in the SRA repository, Bioproject No. PRJNA778616. One extraction reagent control was set in which the different DNA extraction and amplification steps were performed using the same conditions as for the samples but using water as template.

### 16S rRNA Sequences Processing

The analysis of 16S rRNA sequences was performed using QIIME 2 pipeline (v. 2021.4) (57). The sequences in the fastq files were denoised and merged using the DADA2 software (58) as implemented in QIIME 2. The amplicon sequence variants (ASVs) were aligned with q2-alignment of MAFFT (59) and used to construct a phylogeny (60). Taxonomy was assigned to ASVs using a classify-sklearn naïve Bayes taxonomic classifier (61) based on SILVA database (release 132) (62). The taxonomic data tables were collapsed at genus level and filtered excluding taxa with less than 10 total reads and present in less than 30% of samples of each dataset.

### Bacterial Co-Occurrence Networks

Co-occurrence networks were inferred for each experimental condition based on taxonomic profiles. Correlation matrices were calculated using the Sparse Correlations for Compositional data (SparCC) method (63), implemented in the R Studio (64). Network visualization and calculation of topological features and taxa connectedness (i.e., the number of nodes and edges, network diameter, average degree, weighted degree, average path length, modularity, number of modules, average clustering coefficient) was performed using the software Gephi 0.9.2 (65).

### Statistical Analysis

Statistical analysis was performed using R program (version 4.0.4) (66). Differences in oocyst and sporozoite frequency between the groups of infected mosquitoes were compared using Fisher’s exact test with Bonferroni comparison tests. The numbers of oocysts formed in mosquito midguts were compared between infected groups by the Mann-Whitney U test. Differences in relative Ab levels (i.e., OD) among groups of immunized birds in the different time points were compared using two-way ANOVA with Bonferroni multiple comparison tests applied for individual comparisons. Microbial diversity analyses were carried out on rarefied ASV tables, calculated using the q2-diversity plugins. The alpha diversity (richness and evenness) was explored using Faith’s phylogenetic alpha diversity index (67) and Pielou’s evenness index (68). Differences in α-diversity metric between groups were assessed using Kruskal-Wallis test (alpha= 0.05). Bacterial β-diversity was assessed using the Bray Curtis dissimilarity (69) and compared between groups using the PERMANOVA test. Betadisper function was used for the construction of PCoA plot, and an ANOVA test was used to compare the dispersion of the samples by groups. The differential features were detected by comparing the log2 fold change (LFC) using the Wald test as implemented in the compositional data analysis method DESeq2 (70). The number of shared co-occurring taxa among different experimental groups was done in R studio using the package “Venn”.

### Differential Network Analysis

Comparison of the similarity of the most central nodes between two networks was done with the package “NetCoMi” (71) in R studio using the read count taxonomic tables. “Most central” nodes are defined as those nodes with a centrality value above the empirical 75% quartile. The comparison returns Jaccard’s indexes for each of four local measures (i.e., degree, betweenness centrality, closeness centrality, eigenvector centrality) of the sets of most central nodes as well as for the sets of hub taxa between the two networks compared. Thus, the Jaccard’s index express the similarity of the sets of most central nodes as well as the sets of hub taxa between the two networks. Jaccard index of 0 indicates completely different sets while a value of 1 indicates equal sets of most central nodes or hub taxa between the compared networks. The two *p*-values P(J ≤ j) and P(J ≥ j) for each Jaccard’s index are the probability that the observed value of Jaccard’s index is ‘less than or equal’ or ‘higher than or equal’, respectively, to the Jaccard value expected at random, which is calculated taking into account the present total number of taxa in both sets [based on Real and Vargas (72)].

## Data Availability Statement

The datasets presented in this study can be found in online repositories. The names of the repository/repositories and accession number(s) can be found below: https://www.ncbi.nlm.nih.gov/sra, PRJNA778616.

## Ethics Statement

All procedures were performed at the Nature Research Centre in Vilnius, Lithuania, according to Lithuanian and International Guiding Principles for Biomedical Research Involving Animals (2012). Infection experiments and other procedures were reviewed and approved by the Lithuanian State Food and Veterinary Service, Ref. No. 2020/07/24-G2-84. The assessment of the animal health and all described procedures were implemented by trained professionals (under licenses 2012/02/06-No-208, and 2016/01.29-No-344).

## Author Contributions

AC-C and VP conceived the study. JA, RŽ, JM, EP, LM-H, and VP performed the experiments and acquired the data. JA, AW-C, and DO analyzed the data. JA, AW-C, and AC-C prepared the figures. AC-C, VP, and JM contributed reagents and other resources. AC-C, VP, and DO supervised the work. JA, AW-C, AC-C, and VP drafted the first version of the manuscript. All authors contributed to the article and approved the submitted version.

## Funding

This research was funded by the French Government’s Investissement d’Avenir program, Laboratoire d’Excellence “Integrative Biology of Emerging Infectious Diseases” (grant no. ANR-10-LABX-62-IBEID). Funding for VP from European Social Fund (project No 09.3.3-LMT-K-712-01-0016) under grant agreement with the Research Council of Lithuania (LMTLT). AW-C was supported by Programa Nacional de Becas de Postgrado en el Exterior “Don Carlos Antonio López” (Grant No. 205/2018).

## Conflict of Interest

The authors declare that the research was conducted in the absence of any commercial or financial relationships that could be construed as a potential conflict of interest.

## Publisher’s Note

All claims expressed in this article are solely those of the authors and do not necessarily represent those of their affiliated organizations, or those of the publisher, the editors and the reviewers. Any product that may be evaluated in this article, or claim that may be made by its manufacturer, is not guaranteed or endorsed by the publisher.

## Supplementary Material

Supplementary Figure S1 Impact of anti-microbiota vaccines on fecal microbiota of birds. Comparison of ASV richness and evenness, measured with **(A)** Faith’s phylogenetic diversity index and **(B)** Pielou’s evenness index, respectively, among the microbiota of mock-immunized, *E. coli* BL21-immunized and *E. coli* O86:B7-immunized birds.

Supplementary Figure S2 Number of shared co-occurring taxa of *Escherichia-Shigella* among the sub-networks of different experimental group. Venn diagram showing the number of bacteria that are common or unique among the taxa that co-occur directly to *Escherichia-Shigella* in the different experimental group.

Supplementary Table S1 Jaccard indexes of local centrality measures. Jaccard’s indexes for each of local centrality measures (i.e., degree, betweenness centrality, closeness centrality, eigenvector centrality and hub taxa) of the sets of most central nodes for pairwise network comparisons. The two *p*-values, P(J ≤ j) and P(J ≥ j), for each Jaccard’s index were added.
