# Supplementary figures and images for: Anti-Microbiota Vaccine Reduces Avian Malaria Infection Within Mosquito Vectors
Source: Front Immunol. 2022 Mar 3;13:841835. doi: 10.3389/fimmu.2022.841835 (PMC8928750; doi:10.3389/fimmu.2022.841835)

**A**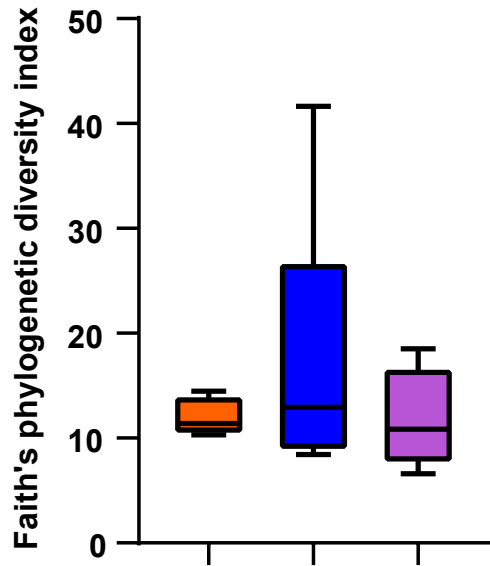**B**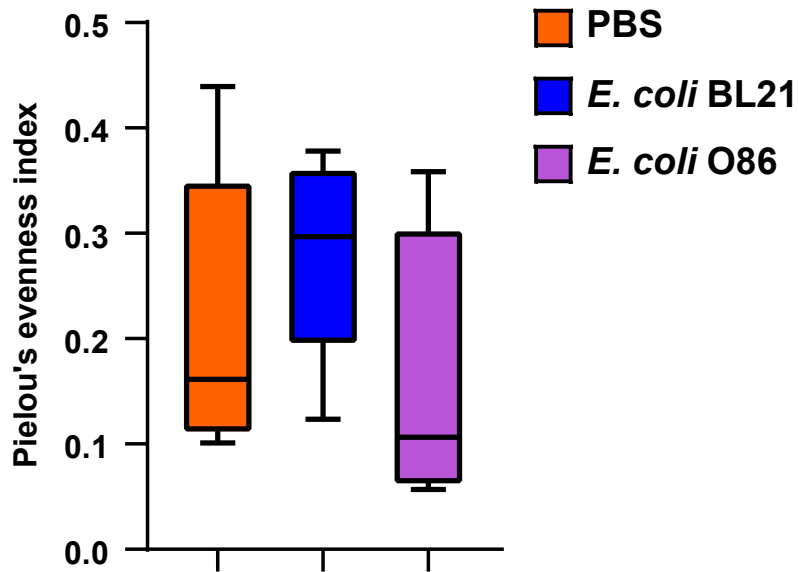

Supplement: Supplementary Figure S1 — Impact of anti-microbiota vaccines on fecal microbiota of birds. Comparison of ASV richness and evenness, measured with (A) Faith’s phylogenetic diversity index and (B) Pielou’s evenness index, respectively, among the microbiota of mock-immunized, E. coli BL21-immunized and E. coli O86:B7-immunized birds. [file DataSheet_1.pdf]

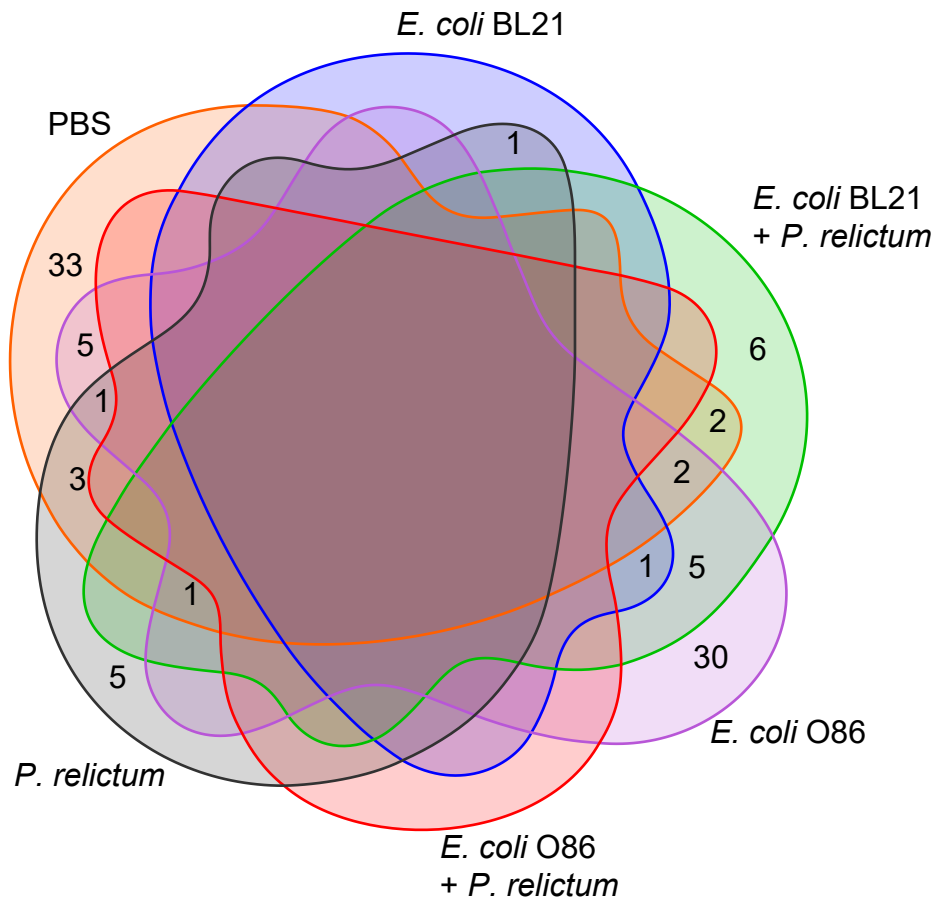

Supplement: Supplementary Figure S2 — Number of shared co-occurring taxa of Escherichia-Shigella among the sub-networks of different experimental group. Venn diagram showing the number of bacteria that are common or unique among the taxa that co-occur directly to Escherichia-Shigella in the different experimental group. [file DataSheet_2.pdf]
